# Novel Method Based on Ion Mobility Spectrometry Combined with Machine Learning for the Discrimination of Fruit Juices

**DOI:** 10.3390/foods12132536

**Published:** 2023-06-29

**Authors:** José Luis P. Calle, Mercedes Vázquez-Espinosa, Marta Barea-Sepúlveda, Ana Ruiz-Rodríguez, Marta Ferreiro-González, Miguel Palma

**Affiliations:** Department of Analytical Chemistry, Faculty of Sciences, University of Cadiz, IVAGRO, ceiA3, Puerto Real, 11510 Cadiz, Spain; joseluis.perezcalle@uca.es (J.L.P.C.); mercedes.vazquez@uca.es (M.V.-E.); marta.barea@gm.uca.es (M.B.-S.); ana.ruiz@uca.es (A.R.-R.); miguel.palma@uca.es (M.P.)

**Keywords:** fruit juices, ion mobility spectrometry, optimization, Box–Behnken, support vector machine, random forest, machine learning

## Abstract

Fruit juices are one of the most widely consumed beverages worldwide, and their production is subject to strict regulations. Therefore, this study presents a methodology based on the use of headspace–gas chromatography–ion mobility spectrometry (HS-GC-IMS) in combination with machine-learning algorithms for the characterization juices of different raw material (orange, pineapple, or apple and grape). For this purpose, the ion mobility sum spectrum (IMSS) was used. First, an optimization of the most important conditions in generating the HS was carried out using a Box–Behnken design coupled with a response surface methodology. The following factors were studied: temperature, time, and sample volume. The optimum values were 46.3 °C, 5 min, and 750 µL, respectively. Once the conditions were optimized, 76 samples of the different types of juices were analyzed and the IMSS was combined with different machine-learning algorithms for its characterization. The exploratory analysis by hierarchical cluster analysis (HCA) and principal component analysis (PCA) revealed a clear tendency to group the samples according to the type of fruit juice and, to a lesser extent, the commercial brand. The combination of IMSS with supervised classification techniques reported an excellent result with 100% accuracy on the test set for support vector machines (SVM) and random forest (RF) models regarding the specific fruit used. Nevertheless, all the models have proven to be an effective alternative for characterizing and classifying the different types of juices.

## 1. Introduction

The production of fruit juices is regulated by the European directive 2012/12 EU, which defines fruit juice as the product derived 100% from the squeezing of healthy and ripe fruit [1]. These beverages are consumed all over the world, as, in addition to their flavor, they are beneficial for health. They are an excellent source of nutrients, especially phenolic compounds and carotenoids. In fact, numerous studies have demonstrated the importance of fruit juice consumption for different functions such as cardiovascular functions [2], cognitive health [3], lipid metabolism [4], etc. In addition to the beneficial properties of juices, they can be consumed throughout the year even though fruit production is seasonal. For these reasons, more and more people are incorporating them into their daily diet [5].

However, fruit juices are one of the products most subject to adulteration, mainly because their main components are water and sugars. For this reason, it is necessary to have adequate analytical tools to characterize the samples and guarantee the quality of the product. In recent years, numerous techniques have been developed for the analysis of fruit juices, such as those based on DNA [6,7], nuclear magnetic resonance [8,9], or spectroscopic techniques such as NIRs [10,11] or FT-IR [12,13]. However, liquid chromatography (LC) and gas chromatography (GC) are among the most widely used techniques. In this sense, liquid chromatography has been successfully used for the characterization of fruit juices [14], as well as for the detection of other adulterations [15,16]. Similarly, GC has also been used to characterize and detect various adulterations in fruit juices [17,18]. Although these techniques offer excellent results, there are some drawbacks such as long analysis times, cost, and the use of solvents.

For this reason, other alternatives, such as headspace–gas chromatography–ion mobility spectrometry (HS-GC-IMS) techniques, have emerged. However, HS-GC-IMS has been scarcely applied in the analyses of fruit juices. Recent studies have demonstrated the potential of this technique for monitoring the fermentation process of sour cherry juice [19] as well as for the differentiation of sweet melon juices according to the fermentation process applied [20]. In addition, it has also been used to analyze flavor and aroma during storage for both bayberry juice [21] and strawberry juice [22].

In terms of data analysis, all these studies are based on the identification of target compounds. This approach has major limitations, such as the complexity of automating the process. Furthermore, in complex matrices such as juices, the differences between different spectra are sometimes very subtle, so identifying of a small number of markers can be difficult and unsuccessful for discrimination purposes.

In this context, the ion mobility sum spectrum (IMSS), obtained through HS-GC-IMS analysis, shows a great potential for food characterization and quality control. This is achieved by combining it with machine-learning algorithms. IMSS has been successfully applied to characterize and detect adulterations of different foods such as honey [23,24], oil [25,26], or coffee [27]. However, to the authors’ knowledge, it has never been used to characterize fruit juices.

Among the most commonly used machine-learning algorithms are exploratory techniques such as principal component analysis (PCA) and hierarchical cluster analysis (HCA). Regarding supervised analysis, parametric techniques such as linear discriminate analysis (LDA) are the most used techniques for classification purposes, and partial least squares (PLS) for regression purposes.

In specific scenarios, when there is no apparent correlation between the spectra and the response variable or when the response variable exhibits non-linearity, the application of more complex non-parametric algorithms becomes essential. Support vector machines (SVM) and random forest (RF) models are notable examples of these techniques. Both SVM and RF can be applied for classification and regression purposes, and they have shown excellent results in the characterization of different fruit juices using different spectroscopic data [13,28,29,30].

Therefore, this study aims to evaluate the feasibility of IMSS in combination with different machine-learning algorithms for the discrimination of different types of fruit juices. For this purpose, an optimization of the most important conditions in the creation of the HS was performed for subsequent analysis by HS-GC-IMS. Finally, both classical parametric techniques (LDA) and more complex non-parametric techniques (SVM and RF) were evaluated and compared.

## 2. Materials and Methods

### 2.1. Juice Samples

Four types of pure fruit juice were selected, and the distribution of the samples was as follows: (I) 9 samples of apple juice (3 brands and 3 batches of each brand), (II) 9 samples of pineapple juice (3 brands and 3 batches of each brand), (III) 9 samples of grape juices (3 brands and 3 batches of each brand), and (IV) 11 samples of orange juice (4 brands, 3 of them with 3 batches and one with 2). All the samples were taken from local markets. Different brands (marketed both nationally and internationally) and different batches were selected to increase heterogeneity within the same type of juice. In addition, each one of the samples was analyzed in duplicate, so the total number of samples was 76. All samples were named by the first initial of the juice used (“O” for orange, “P” for pineapple, “A” for apple, and “G” for grape) followed by the first two initial letters of the brand (“CA”, “DS”, “BI”, “ZS”, “HC”, “JV”, or “GR”) and the batch number (“1”, “2”, or “3”). Furthermore, the number of the replica was indicated with “R1” or “R2”. In this way, an orange juice of the HC brand belonging to batch 2 would be labeled as O_HC_3_R1 for the first replica and O_HC_3_R2 for the second replica.

### 2.2. HS-GC-IMS Analysis Acquisition

The samples were directly analyzed by using the FlavourSpec system (G.A.S., Dortmund, Germany). This system has a static headspace (HS) creation system followed by a gas chromatography column (GC) and an ion mobility spectrometry detector (IMS). The GC column was multicapillary MCC OV-5 (5%-diphenyl, 95%-dimethylpolysiloxane) of 20 cm length and 0.2 µm diameter. The drift and carrier gas selected was nitrogen at 99.999% purity. The HS conditions (sample volume, temperature, and incubation time) were optimized as it is described in Section 2.2.1. The IMS conditions were set based on previous literature in similar samples with some minor modifications [31]. The range of HS conditions studied encompassed sample volume of 250 to 750 µL, temperatures ranging from 40 to 80 °C, and the incubation time from 5 to 15 min. The final conditions of HS and GC-IMS are summarized in Table S1 (Supplementary Material).

In this case, the injection volume was kept at a minimum of 100 µL to avoid possible saturation of the equipment. In addition, to ensure no cross-contamination between the analyses of each sample, a blank (empty vial) was introduced and the syringe was flushed with a stream of nitrogen for 5 min after each injection.

#### 2.2.1. Box–Behnken Design (BBD)

Based on the previous experience and the literature [31], the most influential variables in the HS generation for HS-GC-IMS were optimized: sample volume (range from 250 to 750 µL), temperature (range from 40 to 80 °C), and incubation time (range from 5 to 15 min). To determine the optimal conditions in the HS, a response surface methodology (RSM) was used, specifically, a Box–Behnken design of experiment (BBD). BBD allows the distinction of the independent variables, which are considered a factor with 3 levels, generally coded as −1, 0, and 1, corresponding to the minimum, medium, and maximum values, respectively, and the dependent variable or response. This type of design has the advantage of reducing the number of experiments while evaluating the statistical significance of the effects of the factors, as well as their interaction using mathematical models [32]. 

As mentioned above, the variables to be optimized were as follows: sample volume, temperature, and incubation time. As can be seen in Table 1, the minimum value of the incubation temperature (coded as −1) was 40 °C and the maximum value (coded as 1) was 80 °C. Therefore, the mean value (coded as 0) was 60 °C. In the case of the sample volume, the minimum value was 250 µL, and the maximum was 750 µL. Finally, 5 min was chosen as the minimum value for the incubation and 15 min as the maximum.

The response variable used for the optimization study was the Manhattan distance in the IMSS between samples of two different juices (orange and pineapple). In this way, it ensured that the conditions employed allowed the greatest separation between the groups. In addition, 3 center points were added to the experimental design, with a total of 15 experiments for each juice. Therefore, the total number of samples analyzed was 30. All the experiments were randomly carried out. The conditions used, as well as the value of the response variable (Y_MD_), are summarized in Table S2 (Supplementary Material). 

The RSM model was applied to the results obtained from the BBD design; specifically, a second-order polynomial function was used. Therefore, the correlation between the independent variable and the dependent variables is given by the following Equation (1):

(1) $$Y=\beta_{0}+\sum_{i=1}^{k}(\beta_{i}X_{i}+\beta_{ii}X_{i}^{2})+\sum_{i<j}^{n}\sum_{i=1}^{k}\beta_{ij}X_{i}X_{j}+r$$

where Y represents the response variable; $\beta_{0}\;$ is the modal constant; *βi* corresponds to the main effect coefficient; *βii* represents the quadratic factor coefficient indicating the surface curvature, and the coefficient *βij* represents the interaction between variables *i* and *j*. Lastly, *r* represents the residual value due to random error. The evaluation of the model’s fitness was based on three metrics: the lack-of-fit value, determination coefficient (R^2^), and statistical significance, which were determined through an analysis of variance (ANOVA).

### 2.3. Data Analysis

#### 2.3.1. IMS Sum Spectrum Acquisition

Ion Mobility Sum Spectrum (IMSS; IM Sum Spectrum) was used, which has already been successfully applied for similar purposes [23,27]. Therefore, no chromatographic data are used and the IMSS for all the samples were directly obtained by Laboratory Analytical Viewer software (LAV) (G.A.S., Dortmund, Germany). The spectroscopic range used comprises a total of 881 drift times, ranging from 1020 ms to 1900 ms (relative to RIP).

#### 2.3.2. Multivariate Analysis

The data were stored in two-dimensional arrays (D_nxp_) where *n* refers to the number of samples and *p* to the number of variables (drift times); i.e., the final complete array was D_76x881_. All analyses were performed using RStudio software version 4.2.2 (RStudio Team 2022, Boston, MA, USA). The *rsm* package [33] was used to adjust the BBD design and the RSM. Most of the graphical representations were carried out with *ggplot2* [34] and the HCA was performed with the *stats* package [35]. Finally, classification models (LDA, SVM, and RF) were created using the *caret* package [36].

## 3. Results and Discussion 

### 3.1. Optimization Study

As there were no previous studies related to the analysis of juices by HS-GC-IMS, the first step was to optimize the HS conditions (sample volume, temperature, and incubation time) in order to maximize the IMSS differences among the juices based on their volatile organic compounds (VOCs) profile. For this purpose, a BBD (Section 2.2.1) was carried out and a second-order polynomial equation was fitted as the RSM. The Manhattan distance between the IMSS from samples of two different juices (orange and pineapple) was used as the response variable (the values are summarized in Table S2 of Supplementary Material). Table 2 shows the coefficients, error, and *p*-values resulting from the estimation of this equation. 

As can be seen, the variables that were influential in the model at the 95% confidence level (*p*-value less than 0.05) were: the temperature, volume, and the quadratic effect of temperature. The results show a very good regression between the difference of the spectroscopic data and the variables in the experimental design, with a very high quadratic coefficient of regression (R^2^ = 0.981). In addition, the lack of fit test was not significant (*p*-value = 0.2347), indicating that the model obtained fits the data well. Therefore, it can be concluded that the approximation of the curvature in the response variable by this model was adequate. In this way, the complete Equation (2) was as follows:

Y_MD_ = 6.3332 − 2.0942∙X_1_ + 0.5031∙X_2_ − 0.283∙X_3_ + 0.0034∙X_1_X_2_ − 0.2145∙X_1_X_3_ − 0.2246∙X_2_X_3_ − 1.5351∙X_1_^2^ − 0.2111∙X_2_^2^ + 0.0643∙X_3_^2^ (2)

where Y_MD_ represents the response variable (Manhattan distance in the IMSS) and X_1_, X_2_, and X_3_ correspond to temperature, volume, and time, respectively, representing the three optimized variables. In accordance with the results, a new model was fitted using only the coefficients that had been found to be significant. The new model also shows a correlation between the response and the experimental variables with a quadratic coefficient of regression (R^2^ = 0.956) and also a non-significant lack of fit (*p*-value = 0.512). Therefore, the second-order polynomial equation is defined only by the three significant variables, giving results similar to those obtained with the full equation. The new reduced Equation (3) was as follows:

Y_MD_ = 6.333 − 2.0942∙X_1_ + 0.5031∙X_2_ − 1.5351X_1_^2^ (3)

**Figure 1:**
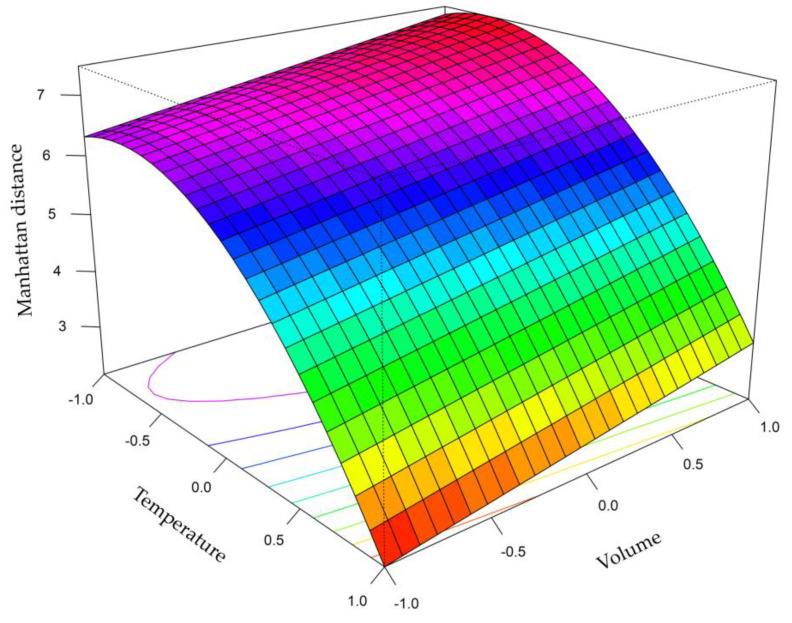
Representation of the effect of the variables, after the adjustment of the second-order polynomial equation.

As can be seen in the model representation (Figure 1), temperature was the most important variable in the model performance, and its curvature is given by the quadratic term. In this case, the response variable (juice separation) decreases dramatically as the temperature exceeds the coded value of −0.687 (optimum point). Theoretically, increasing the temperature would be expected to lead to a greater extraction of VOCs and a thus greater separation between groups. However, it should be noted that excessive temperatures may lead to further degradation and/or fragmentation of the compounds causing the differences to decrease. Based on these results, the optimum temperature was 46.3 °C.

Regarding sample volume variable, it was observed that the response variable increased the greater the amount of sample introduced, and a local optimum has not been obtained in the range studied. However, the sample volume cannot exceed 750 µL because a higher volume of liquids in the sampling vial may cause the needle to reach the liquid during the sampling step for the HS. Therefore, 750 µL was, finally, the sample volume selected for further analysis.

The incubation time in the range studied has turned out to be a non-significant variable, so it has been decided to keep 5 min as the heating time since this is the minimum time to ensure that the HS is homogeneous and reproducible.

Therefore, the optimal conditions for HS generation were as follows: 750 µL sample volume, 46.3 °C incubation temperature, and 5 min incubation time.

### 3.2. Exploratory Analysis

Once the most important variables of the HS were optimized, the samples were analyzed with the conditions summarized in Table S1 (Supplementary Material). For each sample, the IMSS was obtained, and the clustering trend was evaluated. Since one of the main objectives was to determine the ability of the HS-GC-IMS technique to characterize and differentiate the types of juices, a critical preliminary step was to determine if there were differences in the IMSS between the different types of fruit juices used in this study. For this purpose, all juice samples were used, and the clustering trend was checked by means of an unsupervised technique known as hierarchical cluster analysis (HCA).

**Figure 2:**
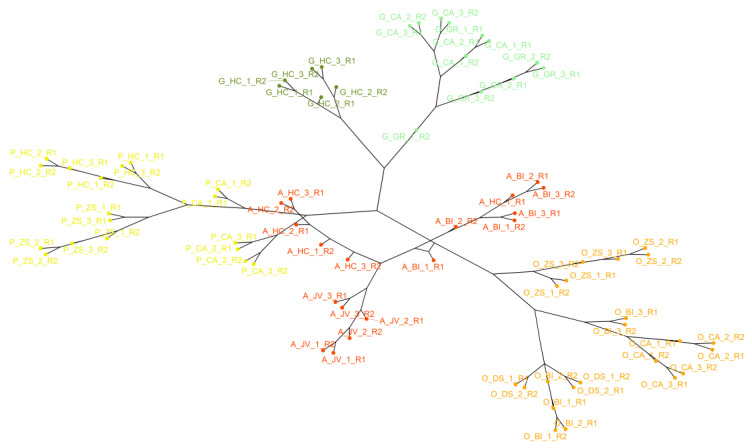
Dendrogram resulting from the HCA analysis using the Manhattan distance and the average method for all IMSS juice samples (D_76x881_). The samples are colored according to the five principal clusters.

In HCA, the Manhattan distance and the average method were used. The choice of this method was based on using the one that had the highest correlation coefficient between the cophenetic distance of the dendrogram and the original distance matrix. In this way, the following methods were tested: single, complete, average, Ward, and centroid, whose values are shown in Table S3 (Supplementary Material). The best result was obtained for the average method (0.9486). Thus, the resulting dendrogram (Figure 2) reflected very closely the true similarity between the observations. This dendrogram was represented in a phylogenetic tree for easier comprehension.

As can be seen, the samples were colored according to the five main existing clusters. Each of them corresponds to a type of juice, except for grape, which comprised two of these branches (dark and light green in the dendrogram). It is observed that apple juice samples (colored in red) were close to pineapple samples (colored in yellow) and orange samples (colored in orange). However, the grape juice samples seem to be the most different (colored dark and light green). Moreover, these samples constitute two independent clusters: one cluster contains the samples of the “GR” and “CA” brands while the other cluster comprises only samples of the “HC” brand. This fact also demonstrated the strong tendency of grouping the samples according to the brand. This tendency was also observed, although to a lesser extent, in the other types of juices. For example, in the case of pineapple (colored in yellow), three almost perfect sub-branches are observed, each one of them containing a single brand.

Based on the HCA, grape juice was the most different from other fruit juices based on the volatile profile. This indicated a greater difference in IMSS and, consequently, in the VOCs that make it up. The other types of juices showed more similarity with each other, especially the apple juice, which sometimes was very close to the orange and pineapple juices. These results indicated that the main clustering trend in the IMSS from the different juices was based on the raw material (type of fruit in the juice), followed by the brand influence that generally divided the main branch into sub-branches. Therefore, the volatile profile obtained by HS–GC–IMS could be useful for fruit juice quality control. 

**Figure 3:**
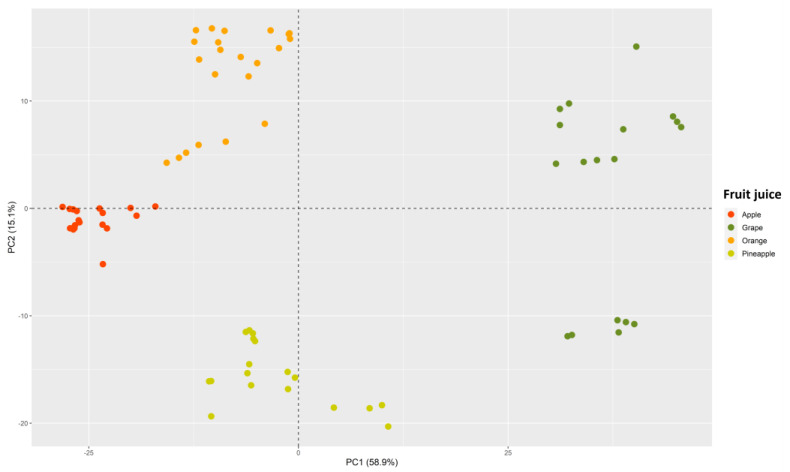
Scores obtained for all IMSS juice samples (D_76x881_) by the first two principal components (PC1 and PC2).

In addition, to corroborate this grouping trend, a principal component analysis (PCA) was performed. Figure 3 represents the scores obtained for the samples in the first two principal components (PC1 and PC2). It is noteworthy that PC1 accounts for 58.9% of the data variability, while PC2 explains 15.1%. Thus, the graph adequately represents the distribution of the juice samples, covering 74% of the available information.

However, to enhance understanding, an interactive plot has been created, which can be accessed via the link https://rpubs.com/JoseLuisPCalle/1051330 (accessed on 1 June 2023). This tool offers greater versatility and includes a third dimension (PC3) that explains 13.1% of the data variability. The graph allows for dynamic rotation to facilitate the viewing of the sample distribution, and, by hovering the cursor over the samples, displays their names. Furthermore, clicking on the legend allows for group removal, providing a closer analysis of each group.

Regardless of the plot, both showed a tendency for samples to group according to the type of juice used. Again, the most different group is the grape juice, obtaining the highest scores for PC1. Furthermore, it is noteworthy that PC2 divided this group into subgroups, with negative values associated with the “HC”-brand grape juices, while positive values were found for the “CA” and “GR” brands. Moreover, a clustering trend was observed for the remaining juices. Orange samples showed positive values for PC2 and negative values for PC1, allowing for their differentiation from pineapple samples, which showed negative values for PC2 and values close to 0 for PC1. Additionally, apple juice samples had negative values for PC1 and values close to 0 for PC2.

Thus, similar to the results obtained with HCA, the samples tended to cluster according to the type of juice analyzed. However, it is important to note that, while satisfactory results were obtained in both HCA and PCA, these analyses do not allow for the perfect classification and prediction of future observations. Therefore, the use of supervised classification techniques is necessary for the accurate classification and characterization of future samples.

### 3.3. Classification Models

The classification models were applied to the complete data matrix (D_76x881_). This matrix comprised 881 drift times and 76 samples, including samples of four juice types (orange, pineapple, apple, and grape). The classification models evaluated include both parametric (LDA) and non-parametric techniques (SVM and RF). 

In this case, the aim was to discriminate the type of fruit used for the juice based on differences in their VOCs profile present in the IMSS. Therefore, four groups were established a priori: “apple”, “orange”, “pineapple”, and “grape”. The data split was randomly carried out and consist of 75% for the training set to create the model and the remaining 25% for the test set to evaluate the performance of the model. Furthermore, it was ensured that the split of both sets was balanced, so the test set contain samples from all type of fruit juice in proportion to the training set.

#### 3.3.1. Support Vector Machine (SVM) with Radial Basis Function (RBF)

SVM is a supervised method based on the concept of hyperplanes. The hyperplane is used to separate the observations, but the separation is not always perfect, allowing for some misclassifications, which are known as support vectors. To control the number of allowed misclassifications, a hyperparameter called Cost (*C*) is used. The value of *C* determines the balance between bias and variance. Additionally, SVM is combined with a radial basis function (RBF), which allows for non-linear separation limits. Therefore, a new hyperparameter, gamma (*γ*), is introduced to control the behavior of the Gaussian kernel, which, in turn, controls the model flexibility [37].

**Figure 4:**
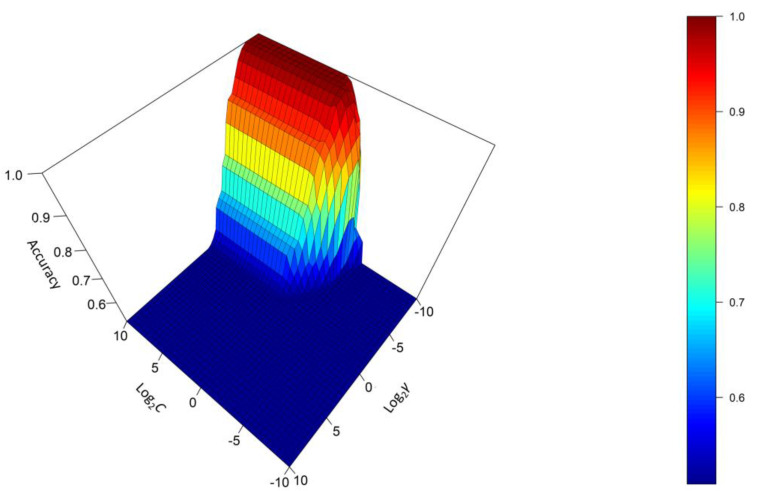
Search for the best combination of hyperparameters (*C* and *γ*) for the Gaussian SVM model with 5-fold cross-validation using the IMSS of the training set samples.

To optimize the SVM model, a five-fold cross-validation was performed using a grid search method with exponentially growing *C* and γ sequences. The values of *γ* and *C* were in the range of [−10, 10] and were taken every 0.5, using log_2_*γ* and log_2_*C*. In five-fold cross-validation, the training data set was divided into five subsets of equal size, where four of these subsets were used to train the model, and the remaining one was used as a test set. This process was repeated for each of the subsets. In total, 8405 models were trained (41 × 41 combinations of *C* and *γ* × 5 subsets). Figure 4 shows the log_2_*γ* values (y-axis) versus the log_2_*C* values (x-axis) and the corresponding accuracy (z-axis) obtained from the cross-validation.

As can be seen, the accuracy reaches its maximum for values of *γ* approximately below 0.031 (log_2_*γ* = −5) and values of *C* above 1 (log_2_*C* = 0). Lower values of *γ* yielded the best results, suggesting that the groups were linearly separable. The optimal *γ* value was set at 0.0028 (log_2_*γ* = −8.5). On the other hand, accuracy increased with higher values of *C*, indicating that the hyperplane will allow fewer misclassified observations and resulting in a less biased model but with higher variance. The optimal value of *C* selected in the optimization was 1 (log_2_*C* = 0).

With the selected combination of hyperparameters, the model achieved a 100% correct classification rate during the cross-validation. Furthermore, the model was able to correctly predict 100% of the samples for both the training and test set. Based on the above, it can be stated that the SVM model with the selected hyperparameters is highly accurate and able to effectively discriminate between the different types of fruit juices based on their VOCs profiles.

#### 3.3.2. Random Forest (RF)

Prior to training an RF model, the number of trees (*ntree* hyperparameter) that make up the model and the value of *mtry* must be chosen. On the one hand, the number of trees must be large enough for the error to stabilize, so, to determine the number of trees to be used, the *ntree* values in this study were checked from 2 to 100 at a two-tree interval. On the other hand, the *mtry* controls the number of variables evaluated before the division of each tree and, in classification problems, it is recommended to use the square root of the total number of variables [37]. Therefore, it was set at 30 (881 variables). Again, the optimization of the *ntree* hyperparameter was performed by five-fold cross-validation. The result is shown in Figure S1 (Supplementary Material) where it was observed that, with six trees, the accuracy reached 100%. However, it was decided to create the model with 30 trees, since there was no high computational cost, and the error was stabilized.

The result of the application of this model showed 100% accuracy in both the training set and test set. Therefore, this model, like the SVM, can be used to discriminate among the different type of juices.

#### 3.3.3. Lineal Discriminant Analysis

In the LDA model, there are no prior hyperparameters to optimize. Therefore, the model was created directly using the training set itself. In this case, 100% correct classification was obtained for the training set and 94.12% for the test set. Only one sample was misclassified, namely, an orange juice that was predicted as an apple. This misclassification may be because, unlike RF and SVM, this model generates linear separation boundaries. In fact, as observed in the exploratory analysis, in both HCA and PCA, the orange and apple juice samples were very close to each other. In order to gather more specific information about the misclassified sample, the LDA model was utilized to obtain the canonical discriminant functions. In this particular case, 4 groups were established beforehand, leading to the obtention of three functions (LD1, LD2, and LD3), which, respectively, accounted for variances of 66.32%, 21.30%, and 12.38%.

**Figure 5:**
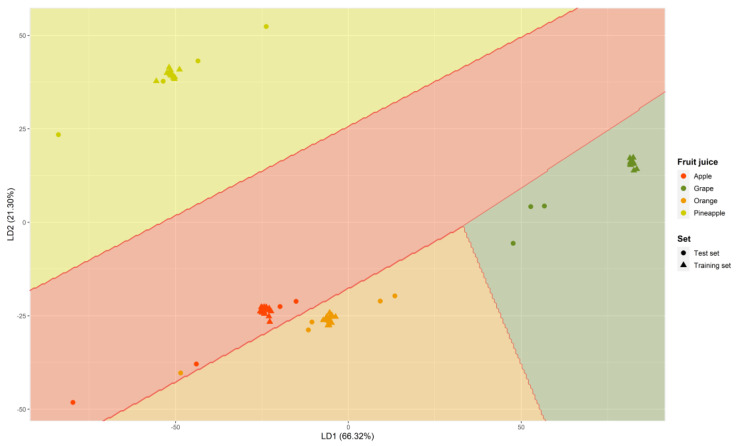
Partition plot for the fruit juice samples (training and test set) obtained by LDA using the IMSS (D_76x881_). The samples have been plotted for the first two canonical functions obtained (LD1 vs. LD2).

The samples were visualized in a partition plot, showing the combination that encompasses the greatest variance (LD1 versus LD2), as depicted in Figure 5. In addition, to facilitate its understanding, the samples have been colored according to the group to which they belong: pineapple juice (yellow), apple juice (red), grape juice (green), and orange juice (orange). Also, shapes have been used to indicate whether the samples belong to the training set (labeled with “▲”) or to the test set (labeled with “●”).

Figure 5 reveals that a single orange juice sample was misclassified and fell within the apple region. This misclassification aligns with that previously discussed, as LD1 and LD2 carry the highest explained variance in the LDA classification. It is also observed that the samples belonging to the test set were the ones that present more variability in their prediction.

Although the performance of the LDA model was satisfactory, it was lower than the SVM and RF models. Therefore, the use of this one would be discarded. It should be noted that other authors, such as Mansour Rasekh et al., reported similar results, with superior performance of the SVM model compared to LDA, for the detection of adulterations in fruit juices using data from an electronic nose [38]. In contrast, other studies have reported better performance with SVM models compared to RF and LDA when using NIR spectroscopic data [28], while, using FT-IR data, SVM and LDA models outperform RF [13].

## 4. Conclusions

To the authors’ knowledge, this is the first time IMSS in combination with ML is applied for the characterization of juice samples from different raw materials. This study successfully optimized the key conditions for creating HS using a BBD design coupled with RSM. The incubation temperature (46.3 °C) and the volume of the sample (750 µL) were the most influential variables. Exploratory techniques like HCA and PCA, combined with IMSS, revealed a tendency to classify the juice samples according to the raw material (type of fruit in the juice) and, to a lesser extent, the brand used. The combination of IMSS with supervised machine-learning algorithms allowed for the accurate characterization and classification of the samples according to the type of juice. The non-parametric models (SVM and RF) reported an accuracy of 100%, outperforming the parametric model (LDA), which achieved an accuracy of 97.76%.

To summarize, the results obtained in the present study demonstrate the potential of IMSS in combination with machine-learning algorithms for the characterization of fruit juices. Moreover, this methodology provides a promising alternative to traditional methods of sample interpretation and analysis, being faster, cheaper, cleaner, and more objective than other traditional methods.

## Figures and Tables

**Table 1:** Values of the levels for the variables used in the experimental design.

Variable	−1	0	1
Incubation time (min)	5	10	15
Incubation temperature (°C)	40	60	80
Sample volume (µL)	250	500	750

**Table 2:** Estimated coefficients of the second-order polynomial equation with their corresponding *p*-values. The interaction is represented by “:” and the quadratic term is indicated by a “^2^”.

Effect	Estimated Coefficient	Standard Error	*p*-Value
Intercept	6.3332	0.2471	<0.001
Temperature	−2.0942	0.1513	<0.001
Volume	0.5031	0.1513	0.0209
Time	−0.2830	0.1513	0.1203
Temperature:Volume	0.0034	0.2140	0.9879
Temperature:Time	−0.2145	0.2140	0.3622
Volume:Time	−0.2246	0.2140	0.3420
Temperature ^2^	−1.5351	0.2227	<0.001
Volume ^2^	−0.2111	0.2227	0.3866
Time ^2^	0.0643	0.2227	0.7843

## Data Availability

The data used to support the findings of this study can be made available by the corresponding author upon request.

## Supplementary Materials

The following supporting information can be downloaded at: https://www.mdpi.com/article/10.3390/foods12132536/s1. Table S1: Conditions used for the analysis of samples using the HS-GC-IMS system. Table S2: BBD design matrix, as well as the experimental values obtained for the response variable (Manhattan distance in the IMSS between juices). Table S3: Correlation between cophenetic distance and distance matrix for different HCA methods using IMSS. Figure S1: RF performance by number of decision trees for 5-fold cross-validation on the training dataset using the IMSS of the samples (D_57x881_).
