# Supplementary material for: Novel Method Based on Ion Mobility Spectrometry Combined with Machine Learning for the Discrimination of Fruit Juices
Source: Foods. 2023 Jun 29;12(13):2536. doi: 10.3390/foods12132536 (PMC10340320; doi:10.3390/foods12132536)
Supplement: Supplementary file 1 [file foods-12-02536-s001.zip › Table S1.pdf]

Table S1. Conditions used for the analysis of samples using the HS-GC-IMS system.

	Variable	Value
HS	Sample volume (µL)	750
	Incubation time (min)	5
	Incubation temperature (°C)	46.3
	Agitation speed (rpm)	750
	Inyection volume (µL)	100
	Syringe filling speed (µL/s)	900
GC-IMS	Syringe temperature	51.3
	Washing time (min)	5
	Inyection speed (µL/s)	850
	EPC1 (mL/min)	250
	EPC2 (mL/min)	2 mL/min (t= 0 min), 10 mL/min (t=5 min), 25 mL/min (t= 10 min)
	System temperature (°C)	51.3
	Column temperature (°C)	55
	Drift tube temperature (°C)	80
Total analysis time (min)	20	
