# Supplementary material for: Novel Method Based on Ion Mobility Spectrometry Combined with Machine Learning for the Discrimination of Fruit Juices
Source: Foods. 2023 Jun 29;12(13):2536. doi: 10.3390/foods12132536 (PMC10340320; doi:10.3390/foods12132536)
Supplement: Supplementary file 1 [file foods-12-02536-s001.zip › Table S2.pdf]

Table S2. BBD design matrix, as well as the experimental values obtained for the response variable (Manhattan distance in the IMSS between juices).

X_1 = Incubation temperature	X_2 = Sample volume	X_3 = Incubation time	Y_1 = Manhattan distance between samples
**-1**	-1	0	5.7598
**-1**	1	0	7.2848
**-1**	0	-1	7.2224
**-1**	0	1	7.0087
**0**	0	0	6.2367
**0**	0	0	6.6418
**0**	0	0	6.1211
**0**	-1	-1	5.9662
**0**	1	-1	6.8961
**0**	-1	1	5.9258
**0**	1	1	5.9574
**1**	1	0	3.4209
**1**	-1	0	1.8823
**1**	0	1	2.0735
**1**	0	-1	3.1450
