# Supplementary material for: Novel Method Based on Ion Mobility Spectrometry Combined with Machine Learning for the Discrimination of Fruit Juices
Source: Foods. 2023 Jun 29;12(13):2536. doi: 10.3390/foods12132536 (PMC10340320; doi:10.3390/foods12132536)
Supplement: Supplementary file 1 [file foods-12-02536-s001.zip › Table S3.pdf]

Table S3. Correlation between cophenetic distance and distance matrix for different HCA methods using IMSS.

Method	Cophenetic Distance
Single	0.9227
Complete	0.9399
Average	0.9486
Ward	0.8239
Centroid	0.9324
