# Supplementary figures and images for: Novel Method Based on Ion Mobility Spectrometry Combined with Machine Learning for the Discrimination of Fruit Juices
Source: Foods. 2023 Jun 29;12(13):2536. doi: 10.3390/foods12132536 (PMC10340320; doi:10.3390/foods12132536)

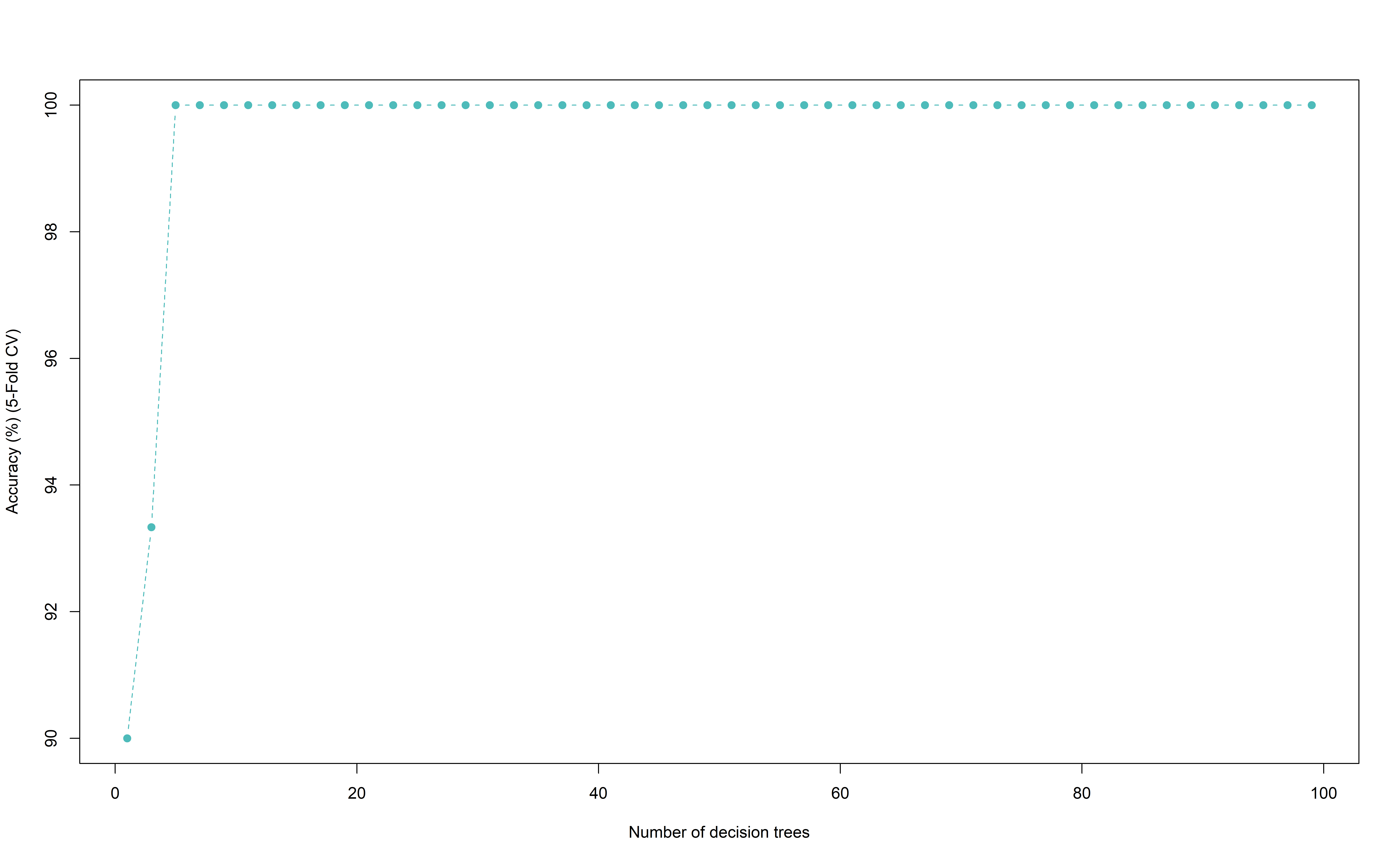

Supplement: Supplementary file 1 [file foods-12-02536-s001.zip › FigureS1.png]
